# Diagnostics of IgE-mediated occupational allergies: Between reality, requirements, and opportunities 

**DOI:** 10.5414/ALX02500E

**Published:** 2024-05-31

**Authors:** Monika Raulf, Sabine Kespohl

**Affiliations:** Institute for Prevention and Occupational Medicine of the German Social Accident Insurance, Institute of the Ruhr-Universität Bochum (IPA), Bochum, Germany

**Keywords:** allergy diagnosis, occupational allergens, high molecular weight allergens, individual prevention, standardization, occupational disease

## Abstract

Occupational skin and respiratory allergies are among the most common occupational diseases in Germany. The identification of the allergy trigger is essential for the recognition of an occupational allergy as well as for effective individual prevention. However, occupational type I allergens are among the “rare” allergens and the possibilities of guideline-compliant diagnosis using quality-tested skin test solutions is becoming increasingly difficult due to the reduction in commercially available test allergens. In order to guarantee meaningful diagnostic workup for all affected insured persons with suspected occupational type I allergies and to ensure this in the future, a durable optimization, standardization, and availability of allergy tests for occupational allergic diseases is urgently required. The need for action has been recognized by the German Social Accident Insurance (DGUV), and steps to eliminate the diagnostic gaps have been initiated by a joint research project at the Institute for Prevention and Occupational Medicine of the DGUV (IPA) and the Paul Ehrlich Institut (PEI). The evaluation of alternative methods for the production of standardized test allergen solutions can also be used for newly emerging allergens in the workplace. New allergen sources at workplaces and thus also sensitization and allergies among employees can be expected as a result of changes in work processes and the introduction of new technologies and/or working materials, which are also introduced in connection with climate change and the concept of sustainability.

## Introduction 

Work-related allergies have been one of the most common occupational diseases for years, affecting both the skin and the respiratory tract. Data from a joint statement by the American Thoracic Society and the European Respiratory Society from 2019 shows that ~ 15% of asthma cases in adults are triggered by substances present in the workplace and that in ~ 20% of adults, pre-existing asthma is exacerbated by workplace conditions [1]. Occupational asthma can be triggered by both irritant and sensitizing substances in the workplace. While occupational asthma caused by irritants can be triggered by acute or prolonged exposure to high or moderate concentrations, allergic occupational asthma induced by sensitizing substances only occurs after a latency period. Allergic diseases of the upper and lower respiratory tract and the lungs are predominantly caused by airborne or aeroallergens. Allergens are rarely absorbed through the skin (e.g., contact urticaria or through injections such as stings). More than 400 agents have now been identified and described as potential occupational triggers (http://www.a​o​ecdata.org/ExpCodeLookup.aspx). The most common triggers are high molecular weight substances – predominantly with a molecular weight between 10 and 100 kD – usually (glyco)proteins, which are contained in flour and grain dust, farm and laboratory animal dust, mites, feed, washing enzymes, molds, natural latex, and wood dust. Low molecular substances such as isocyanates, acid anhydrides, metals, ammonium persulphates, and vapors from washing, bleaching, and fixing agents in the hairdressing sector, disinfectants, cleaning agents, and pharmaceuticals can also have a sensitizing effect. However, the immunological mechanism of action of these low molecular substances has not been clearly understood. 

## Allergies as an occupational disease and changes in occupational disease law in Germany 

**Figure 1:**
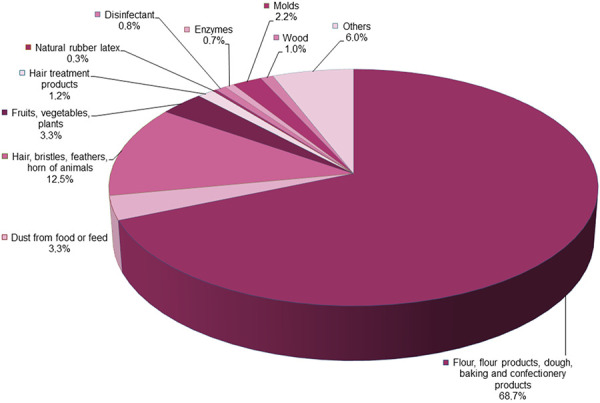
Frequency of the triggering allergen sources of occupational disease BK No. 4301 (“obstructive respiratory diseases caused by allergenic substances including rhinopathy”). Summarized representation of the most frequent triggering allergen sources from the data of the BK-DOK of the German statutory accident insurance institutions and the German Social Insurance for Agriculture, Forestry and Horticulture (SVLFG) for the period 2018 – 2022.

In the German Occupational Diseases Ordinance, the occupational disease (BK) “obstructive respiratory diseases (including rhinopathy) caused by allergenic substances” is classified under the number 4301 (BK No. 4301). Both the industrial employers’ liability insurance associations and public sector accident insurance institutions (information from the BK-DOK of the DGUV 2023) and the Social Insurance for Agriculture, Forestry and Horticulture (SVLFG) record both the reported and the recognized cases of BK No. 4301 in their current occupational disease documentation statistics. As the “BK-specific object”, i.e., the disease-causing noxious agent for the recognized occupational diseases of BK No. 4301 in the period from 2018 to 2022, flours, flour products, dough, and bakery products are clearly prominent with more than 65%, followed by animal allergen sources such as hair, bristles, feathers, horn with 12.5% (Figure 1). 

The underlying mechanism of BK No. 4301 is a type I allergy (immediate-type reaction). Other occupational diseases that are also caused by the inhalation of antigens and can lead to allergic diseases are hypersensitivity pneumonitis (BK No. 4201) and BK No. 1315 “Isocyanates”. The latter includes all isocyanate-related occupational diseases with the exception of skin diseases. Severe or recurrent skin diseases are summarized under BK No. 5101. When considering this occupational disease number, it must be taken into account that both allergic (here, the majority of cases are type IV reactions) and non-allergic skin diseases are recorded together so that it is not possible to consider the allergic event alone [2]. 

In principle, only those diseases that are caused by specific occupational factors and conditions according to the findings of medical science can be considered and therefore recognized as occupational diseases. Certain groups of subjects must be exposed to these effects to a significantly higher degree than the rest of the population due to their occupational activity. For some diseases listed in the Occupational Diseases Ordinance, including BK No. 5101 and BK No. 4301, recognition as an occupational disease requires that the harmful activity that caused or may have caused the development, aggravation, or recurrence of the disease be discontinued. 

Since January 1, 2021, changes to the law on occupational diseases have come into force, which the German Bundestag passed on May 7, 2020, as part of the 7^th^ SGB IV Amendment Act. The adopted amendment to the law on occupational diseases provides, among other things, for the “elimination of the obligation to refrain” as a criterion for the recognition of occupational diseases [3, 4]. With regard to allergic diseases, this resulted in changes for BK No. 4301 and BK No. 5101. The legislator justifies the abolition of the obligation to refrain on the grounds that it was a historical instrument that is now outdated and could certainly be detrimental to the insured person. However, in order to ensure that remaining in employment after the change of the law does not lead to further injury through the continuation of the previous activity, greater emphasis is placed on individual prevention. Here, the insured person is obliged to participate and cooperate in measures designed to prevent the progression of the occupational disease. The available data on the effectiveness of individual prevention measures is so far rather weak [5]. It therefore makes sense to review individual measures by means of accompanying studies. 

In the case of allergic diseases, early identification of the allergen sources causing the symptoms is necessary for the introduction of targeted preventive measures for the employee concerned. This requires targeted diagnostic workup in which a causal relationship between exposure to the working substance and the allergic symptoms is established. Furthermore, reliable diagnosis is of great importance for patients with occupational allergies, as treatment is essentially based on avoiding the allergen source. Furthermore, the earlier the trigger is clearly identified and avoidance (or reduction) of exposure is initiated, the greater the chances of a positive health outcome. In this way, it is possible to avoid having to give up work. 

## Identification of the allergy trigger is essential 

In principle, if an occupational respiratory disease (rhinopathy, obstructive airway disease) is suspected, an allergic pathogenesis should be considered and the connection between the symptoms and the workplace should be proven by means of targeted diagnostics. The difficulties in making a correct diagnosis are not inconsiderable. On the one hand, allergic diseases are common in the general population so that it is often difficult to distinguish between occupational and non-occupational causal factors. On the other hand, additional non-specific factors can play a role in the pathogenesis of allergic diseases and in their progression, especially with regard to the degree of hyperreactivity of the airways. Allergy diagnostics are therefore of central importance. It is therefore all the more important that the diagnosis of occupational allergies can always reflect the current allergen situation at the workplace. This means that relevant allergen sources should be available as test extracts. In individual cases, it is important to assign the symptoms to a clinical picture, to determine the causative allergy trigger, the allergen source, and to diagnose the sensitization reliably so that a targeted and comprehensive allergen avoidance can be initiated. Ultimately, the detection of the allergen causing the symptoms at the workplace is therefore a basic prerequisite for all preventive measures relating to occupational allergies. 

**Figure 2:**
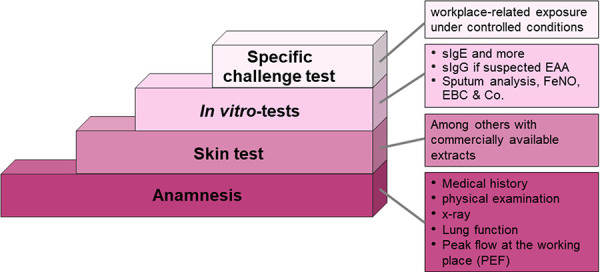
Step-by-step workflow for allergy diagnosis in cases of suspected occupational type I allergies.

Allergy diagnosis is carried out in four steps: patient history – skin tests – in vitro laboratory tests – challenge tests (Figure 2). 

Following a detailed and indicative medical history, skin tests with occupational substances are initially the method of choice both in the dermatologist’s procedure and in the occupational disease assessment procedure. The prick test is the first option for detecting an IgE-mediated immediate-type allergy. The prick test is inexpensive, the results are quickly available, and it is also a sensitive method for detecting sensitization – but only if a standardized methodological procedure with validated and standardized extracts is used. In addition, the test result is visible to the patient and can therefore also have an educational effect. However, it should be noted that a positive skin test reaction as well as a positive detection of specific IgE only indicate sensitization and the causal relationship between occupational exposure and sensitization can usually only be established by a specific inhalation challenge (SIC) test [6]. The SIC is considered the gold standard, especially if the affected persons are no longer working at their original workplace [7]. According to the SIC guideline, it should only be performed in a specialized facility [7]. If workplace-related inhalation challenge testing is not possible in a specialized facility and there is a strong suspicion of occupational asthma based on the medical history, a combination of objective signs of asthma or rhinitis and a positive skin test or the detection of specific IgE for the suspected trigger has a high predictive value for an occupational respiratory disease if it is a high molecular allergen source [8]. Therefore, skin testing or serological evidence of sensitization is highly relevant for the assessment and the associated socio-economic consequences. 

## Quality testing and assurance of diagnostics 

As skin tests are of great importance in the diagnosis of occupational type I allergies in particular, the quality of the available skin test preparations also plays a decisive role in the validity of the diagnosis. In most cases, the allergen test extracts are produced from natural allergen sources and can therefore be variable in their composition. Comparative evaluations of prick test solutions of nominally identical allergen sources from different manufacturers both for occupational allergens [9] and for molds [10, 11] showed clear differences in protein content and in some cases to an even greater extent in antigen content. In general, sensitivity and test efficiency in in vivo testing were higher for solutions with higher protein and antigen content. However, it was found that the level of protein content was not always a meaningful parameter for the quality of a prick test solution. The reason for this was the addition of non-allergenic proteins, such as human serum albumin as a stabilizer in some solutions. Based on the results of the STADOCA project, an EAACI position paper on skin prick testing in the diagnosis of occupational type I allergies was published [12], in which it is recommended to use prick test solutions from different manufacturers in parallel due to the sometimes low sensitivity of some prick test solutions – especially in the case of a negative test – and to carry out the tests in duplicate on the left and right volar side of the forearms if possible. In principle, a standardization of prick test solutions, particularly for occupational allergens, is also required against the background of the importance of qualitatively equivalent diagnostics for all insured persons in a dermatologist’s procedure or an occupational disease assessment procedure. 

In accordance with EU Directive 2001/83/EC, which is implemented in Germany in the German Medicinal Products Act (AMG), allergen test extracts and haptens are medicinal products subject to authorization, as they are used to establish a medical diagnosis. They are subject to batch testing at the Paul Ehrlich Institut [13]. This is certainly an important step towards quality assurance, but it has also led to a significant reduction in the number of available test allergen extracts due to low demand. The active withdrawal of authorizations for test allergens for the detection of occupational immediate-type allergies or the expiry of authorizations due to the so-called “sunset clause” (Section 31 (1) sentence 1 number 1 of the AMG) has created a diagnostic gap. This mainly affects rare test allergens, but also occupational test allergens in particular. Manufacturers cite economic inefficiency as a reason for this, as even with relatively low demand, especially for rare test allergens, the requirements of good manufacturing practice and quality standards must be guaranteed in accordance with the corresponding authorization [14]. Of the original 30 prick test solutions relevant for occupational type I allergies that were used as part of the STADOCA project mentioned above, only 16, i.e., just over half, are currently listed as authorized allergens on the Paul Ehrlich Institut website https://www.pei.de/DE/arzneimittel/allergene/pricktest/pricktest-node.html?cms_gtp=174138_list%253D2%2526174126_list%253D4&cms_tabcounter=5#anchor.

The unavailable test allergens – in relation to the occupational field – are quite common allergens, such as rye flour and bovine epithelia. This loss limits allergy diagnostics in the field of occupational allergens enormously. 

The recommendation of the guideline “Skin tests for the diagnosis of allergic immediate-type reactions” [15] also applies to skin testing, including in the occupational disease recognition procedure for BK No. 4301 and BK No: 5101; this includes that tests should always be carried out with standardized extracts first. However, other test substances can be used if standardized test allergens are not available. In the case of clarification of an occupational cause of the disease, the individualized production of test materials by the doctor for a specific patient is possible without a manufacturing authorization in accordance with Section 13 para. 2b sentence 1 AMG. According to § 67 AMG, however, there is an obligation to notify the competent supervisory authority that individualized tests are being carried out with test preparations that are not authorized as medicinal products. An informal letter with a description of the activity to the competent authority (GCP inspectorates of the federal states; https://www.zlg.de/arzneimittel/deutschland/laenderbehoerden, then choose the federal state) is often sufficient. However, the consequence of this option is that, although the diagnostics are useful on an individual basis, they cannot be quality-assured and standardized and can lead to unequal assessment and ultimately unequal treatment of the employees concerned. 

## Recognizing the need for action and steps to close the diagnostic gap 

In order to guarantee meaningful diagnostics for all affected insured persons with suspected occupational allergies and to ensure this in the future, a permanent optimization, standardization and availability of allergy tests for occupational allergic diseases is urgently required. The German Social Accident Insurance (DGUV) has therefore taken up the need for action and started a research project (Berufsderma 164) at the Institute for Prevention and Occupational Medicine (IPA). Together with the Paul Ehrlich Institut, which is supported by the DGUV research funding (FB 317a) in this cooperation project, it is being investigated how meaningful diagnostics can be guaranteed in the long term for affected insured persons with suspected occupational type I allergies. In addition to efforts to counteract the further reduction in commercially available test solutions (e.g., through exchanges at various levels with manufacturers and, since 2018, in coordination with the Federal Ministry of Health through a reduction in fees charged by the higher federal authority (Paul Ehrlich Institut) to a quarter for all official acts in connection with rare test allergens (e.g., scientific advice, authorization, change notifications, official batch testing, etc.)), this joint feasibility study funded by the DGUV will examine whether the production of prick test solutions in public pharmacies is feasible taking into account all legal and regulatory conditions. 

In a first step at the beginning of this cooperation project, a priority list of the 20 occupationally relevant allergen extracts for the detection of occupational type I allergies that are essential for the care of insured persons was drawn up together with several accident insurance institutions. This priority list includes allergen sources from the following groups: flours, enzymes, storage mites, woods, molds, animal epithelia, natural latex, fish, and seafood (Table 1). Some of these allergens are also relevant beyond the workplace (e.g., storage mites). Based on the results of quality testing of allergen extracts and the requirements for sufficient protein, antigen, and allergen content for sensitive and specific test extracts, the project plan envisages the development or adaptation of standard operating procedures for the production of extracts – if not yet available. Depending on the starting material, individual adaptation of the production steps will be necessary. The characterization of the allergen extracts is carried out both protein-biochemically and immunologically and by comparison with commercial extracts, if available. Based on the established and standardized laboratory procedures for the production of allergen extracts, a methodical adaptation of the extract production in accordance with the AMG to standard pharmacy conditions [16] and possibilities will be investigated. The procedure for the preparation of selected test solutions in a selected pharmacy is described in Kespohl et al. 2024 [17]. Further testing of the prepared test solutions for skin testing on patients is to be carried out in an in vivo skin test validation study of the IPA in cooperation with medical centers and practices. Although the primary aim is still to obtain commercially available test solutions, the results of the study show that the production of selected skin test solutions, especially when protein-rich starting materials are involved, is feasible in selected public pharmacies. This validated the possibility of a “plan B” to ensure long-term diagnostics for occupational type I allergies under standardized conditions. 

## Challenges and perspectives for future allergy diagnostics 

For many ubiquitous allergen sources, the use of component-resolved diagnostics now offers the possibility of clarifying the sensitization profiles of affected patients in detail and thus obtaining information about cross-reactivity and therapy options, among other things. The use of allergen molecules to support the recognition of an occupational allergy is very limited [18]. This is partly due to the small number of characterized occupationally relevant allergen sources, but also to the fact that the sensitization pattern for wheat flour, for example, the most relevant allergen for baker’s asthma, is very complex and varies from person to person, so that no major allergen has yet been identified. In contrast, if a natural latex allergy is suspected, recombinant single allergens can provide targeted and effective support in clarifying the clinical relevance and the trigger for the symptoms [19]. 

Changes in work processes, the introduction of new technologies and/or working materials, but also climate change with its direct and indirect consequences, as well as adaptation strategies and lifestyle changes often lead to new allergen exposures at workplaces and thus also to new sensitization and allergies. For example, employees who work outdoors are exposed to potential allergen sources whose distribution is strongly influenced by climatic changes, such as oak processionary moths, ticks, or molds (e.g. *Cryptostroma corticale*). Lifestyle changes that primarily serve climate protection and sustainability can lead to new products and manufacturing processes and thus also to new sensitizing hazards at workplaces. The sensitizing risk of inhaled enzyme exposures at production workplaces but also in processing and quality control processes should be taken into account [20]. 

For all these new allergen sources, there are still no, or only a few, available test instruments that can be used for allergy diagnosis. It is therefore necessary to pay greater attention in the future to occupational allergen sources, which can also be environmental allergens, such as storage mites. The development processes and changes in the world of work described above should also be taken into account with regard to the occurrence of allergies. Characterization of allergen sources and knowledge of allergenicity are important for risk assessment and can be used to introduce targeted protective measures. Furthermore, quality-assured diagnostics must be ensured in order to enable equal treatment of affected employees. 

## Authors’ contributions 

Idea and conception of the paper as well as writing of the manuscript MR, completion by SK; final version authorised by both. 

## Funding 

This study was financially supported by the German Social Accident Insurance (DGUV) (part of the IPA project 164 Berufsderma and by the DGUV research funding project FB 317A). The funder had no influence on the study design, data collection and analysis, the decision to publish, or the preparation of the manuscript. 

## Conflict of interest 

Both authors declare that there is no conflict of interest in relation to this publication. MR received honoraria for speaking engagements from the following companies and associations between 2020 and 2024: Alk-Abelló Arzneimittel GmbH, Berufsverband Deutscher Baubiologen VDB e.V, Haus der Technik, LetiPharma, ThermoFisher Scientific (Phadia). With regard to the content of this article, there are no conflicts of interest that could arise from an employment relationship, benefits for lectures, or other activities. 

**Table 1.** Priority list* of the 20 priority occupational allergens required for skin prick test solutions.

**Allergen**	**Allergen**
**Wheat flour**	Cattle hair (cattle epithelia)
**Rye flour**	Mouse urine/hair (mouse epithelia)
α-amylase	Rat urine/hair (rat epithelia)
Glucoamylase	***Aspergillus fumigatus***
***Lepidoglyphus destructor***	***Penicillium chrysogenum***
***Tyrophagus putrescentiae***	***Aspergillus versicolor***
***Acarus siro***	*Stachybotris chartarum*
Spruce wood	Phytase (Natuphos)
Beech wood	**Fish (cod)**
Natural rubber latex	**Shrimp**

*Priority list drawn up with representatives of various German accident insurance institutions (UVT). **Bold print:** Allergen source material that is commercially available from certified allergen supplier and can be used for the preparation of allergen extracts according to the Medicinal Products Act, The European Pharmacopoeis and the Pharmacy Operation Regulations.
